# Does owning a bank account improve reproductive and maternal health services utilization and behavior in India? Evidence from the National Family Health Survey 2015–16

**DOI:** 10.1016/j.ssmph.2019.100396

**Published:** 2019-04-06

**Authors:** Abhishek Singh, Kaushalendra Kumar, Lotus McDougal, Jay G. Silverman, Yamini Atmavilas, Raksha Gupta, Anita Raj

**Affiliations:** a International Institute for Population Sciences, Mumbai, India; b Center on Gender Equity and Health, University of California San Diego, San Diego, USA; c The Bill & Melinda Gates Foundation, New Delhi, India

## Abstract

• Ownership of a bank account is associated with improved reproductive and maternal health services utilization and behaviour.
• Observed associations are strongest in states where the utilization of studied services is far below the national average.
• No association is found between women's ownership of a bank account and institutional delivery.

## 1 Introduction

Multi-country research documents that the combination of women's higher economic status and indicators of empowerment (e.g., decision-making control) increase reproductive and maternal health care utilization in low and middle income countries, suggesting the potential value of women's economic empowerment for maternal health (Ahmed, Creanga, Gillespie, & Tsui, 2010). Economic and other social science theories of gender empowerment suggest that women's financial inclusion supports assets and resources that can facilitate women's knowledge of the value of health care, mobility to obtain health services and sense of personal entitlement and self-efficacy to prioritize their health (Cornwall, 2016; Kabeer, 1999, 2005; DAC Network on Gender Equality, 2011; World Bank, 2014). Evaluation studies indicate that women's participation in microfinance can significantly increase reproductive and maternal health services utilization and reduce maternal mortality (Orton et al., 2016), but there has been little examination of other forms of women's financial inclusion in terms of their association with maternal health. This study seeks to examine the association between ownership of a bank account, a growing form of financial inclusion for women, and reproductive and maternal health services utilization and behavior among a nationally representative sample of women in India.

### 1.1 Reproductive and maternal health services utilization and behavior in India

Substantial reduction in maternal mortality in India over the past twenty years has been linked to improvements in maternal health care utilization in this same period (O'Neil, Naeve, & Ved, 2017). India, in the past couple of decades, has taken a major leap in the direction of improving the overall health of the population in general, and the health of poor and marginalized sections of the population in particular. In 2005, the Government of India launched the National Rural Health Mission (NRHM), its most ambitious programme which aimed to improve the overall health of the rural population, as well as to improve the health of women and children in rural areas. Janani Suraksha Yojana (JSY) was identified as one of the strategies directed towards improving the health of the women and children. Under JSY, women were to get financial incentives if they deliver their babies in government medical institutions or in medical institutions accredited by the Government (Government of India, 2005). Later on, in 2011 the Government of India expanded the JSY into Janani Shishu Suraksha Karyakaram (JSSK) to promote compulsory postnatal care for the newborn and the mother, and the early initiation of breastfeeding. Further, there is a provision for free care in case a newborn becomes sick from the time of birth till 30 days after the birth (Government of India, 2011). Most recently, the Government of India expanded the NRHM into an overarching National Health Mission (NHM) which has two sub-components: NRHM and the National Urban Health Mission (NUHM) (Government of India, 2013).

While data from recently released National Family Health Survey (NFHS) 2015-16 suggest significant improvements in coverage of a number of indicators including availing antenatal visits, delivery in a medical facility, the coverage of a number of these indicators are far from universal (IIPS & ICF, 2017). For example, four or more antenatal visits increased from mere 37% in NFHS 2005-06 to 51% in NFHS 2015–16. The percentage of births in medical institutions increased from 39% in NFHS 2005-06 to 79% in NFHS 2015–16. On the other hand, the current use of contraceptives has not changed between 2005-06 and 2015–16. Remember that national averages hide significant socio-economic and residence related inequalities. This is particularly true in a large and diverse country like India. For example, among bigger states, delivery in a medical institution ranges between as low as 62% in Jharkhand and as high as 100% in Kerala (IIPS & ICF, 2017). Important differences by social inequality indicators can also be masked by national prevalence data. For example, while national data indicate that 79% of women who gave birth in the past five years received antenatal care from a clinical provider, this held true for only 75% of rural women, 61% of women without an education, and 57% of women in the poorest quintile (IIPS & ICF, 2017). Similarly, while 79% of births now occur in a health care facility, this holds true for 75% of rural births, 62% of births to women with no education, and 60% of births in the poorest quintile (IIPS & ICF, 2017). These findings demonstrate that despite improvements in reproductive and maternal health service utilization in India, there remains need to reach socially vulnerable women for these services.

### 1.2 Women's financial inclusion and bank account ownership in India

In 40% of nations, women have less access to financial systems than do men (WEF, 2018). India ranks 142 of 149 nations in terms of gender equality in economic participation and opportunity (WEF, 2018). However, one area that India has done quite well is financial inclusion in the form of bank accounts (CRISIL, 2018). Bank accounts, a more accessible form of financial inclusion than microfinance, are increasing in availability; now over half of all adult women globally (58%) have a bank account (Steiner, 2018). In 2014, the Government of India, as part of its National Mission for Financial Inclusion, launched Pradhan Mantri Jan-Dhan Yojana (PMJDY) to support every adult in India to have a bank account inclusive of mobile banking accessible via cell phones (Government of India, 2014). The largest number of beneficiaries of this initiative are expected to be women and the rural poor, populations most affected by low reproductive and maternal health services utilization, and subsequent maternal and infant morbidities and mortality (IIPS & ICF, 2017). From 2011 to 2017, the nation has seen a more than doubling of bank account ownership, and most recent evidence suggests that 80% of adults in India now own a bank account (Demirgue-Kunt et al., 2017). During the period of 2014–2017, this increase was even greater among women, rural and the poorest populations in the country, though women remain disproportionately less represented than men among account owners (Demirgue-Kunt et al., 2017).

### 1.3 Women's bank account ownership and health care utilization

While there is some evidence on the association between microfinance and reproductive and maternal health services utilization and behavior, as well as other positive health outcomes (Hamad & Fernald, 2015; Mohindra, Haddad, & Narayana, 2008; Orton et al., 2016; Schuler & Hashemi, 1994; Steele, Amin, & Naved, 1998), research on the relationship between bank account access or ownership and health outcomes is very limited. An analysis with 176 countries found that gender inequalities in bank account access (i.e., lower access for women relative to men) was associated with higher female to male stroke ratio at the nation level (Kim, Jung, Caso, Bushnell, & Saposnik, 2017), and a study with older Hispanics in the United States found that bank account ownership was associated with improved mental health (Aguila, Angrisani, & Blanco, 2016). The sole study we could identify from India examined the association between bank account ownership and risk for spousal violence; using longitudinal data from married women in Maharashtra, this study found bank account ownership reduced risk for spousal violence (Raj et al., 2018). We could identify no study that has examined associations between women's ownership of a bank account and reproductive and maternal health services utilization and behavior.

This study seeks to expand on the growing literature on women's economic empowerment and health by examining bank account ownership and reproductive and maternal health services utilization and behavior in India. As part of increased government attention and focus on bank account ownership in India, a question on bank account ownership for women was included in the fourth round of the National Family Health Survey (NFHS-4) conducted in 2015–16, newly enabling individual-level, nationally-representative analysis of bank account ownership and health care use associations in India. Timing of NFHS-4 was such that the assessment was just prior to substantial growth in women's bank account ownership, allowing for opportunity of examination of this issue before saturation of bank account ownership.

In the presence of selectivity of women owning a bank account, standard epidemiologic analyses adjusting for demographics and region may yield biased results. Propensity score matching allows us to estimate the effect of ownership of a bank account on our reproductive and maternal health care outcomes, while accounting for underlying differences between women who do and do not own bank accounts. Given the lack of prospective or evaluation data to examine our research question, this analytic approach offers important information regarding the potential value of bank account ownership as a form of financial inclusion that can be used to promote reproductive and maternal health care in India.

## 2 Material and methods

### 2.1 Data

We analyzed data from NFHS-4 conducted in 29 states and 7 Union Territories of India during 2015–16. The main objective of the NFHS-4 is to provide essential data on health and family welfare, and other emerging issues in India. The NFHS-4 adopted a stratified two-stage sampling design in both rural and urban areas. In rural areas, villages were selected in first stage using a Probability Proportional to Size (PPS) scheme. In the second stage, 22 households were selected using systematic sampling. In urban areas, census enumeration blocks (CEBs) were selected in the first stage using PPS scheme. In the second stage, 22 households were selected using systematic sampling. Over 699,000 women age 15–49 were interviewed in NFHS-4, with a response rate of 97% (IIPS & ICF, 2017).

### 2.2 Variables

The reproductive and maternal health services and behavior outcomes included in the analysis are current use of contraceptives, birth spacing (as a validation indicator of effective contraceptive use), availing antenatal care (ANC), and institutional delivery. We coded current contraceptive use into four categories - no method, modern spacing method, modern limiting method, and traditional method. For the analysis of the current contraceptive use, we included the sample of all currently married women age 15–49 who were not pregnant or unsure of pregnancy, and fecund (n = 81,480).

The birth spacing variable was constructed using birth interval data on non-first order births in the past five years. Children born after intervals less than 24 months are at higher risk of mortality and under nutrition compared with their counterparts (Gribble, Murray, & Menotti, 2008; Rutstein 2005, 2008; Rutstein, Johnson, & Conde-Agudelo, 2004). Children born after intervals less than 36 months are also at elevated risk of mortality and under nutrition. Hence, we included two variables for analyzing birth spacing. First, whether the preceding birth interval was less than 24 months or otherwise. Second, whether the preceding birth interval was less than 36 months or otherwise. Both of these variables are binary. Analyses were limited to all higher order singleton births in the past five years (n = 26,276).

Availing ANC is based on most recent birth in the past five years, coded into three categories - No visits, 1–3 visits, and 4 or more visits. We categorized the variable in this way based on WHO and Government of India recommended standards of four or more ANC visits (Government of India, 2010; WHO, 2006). Analyses were limited to most recent singleton birth in the past five years (n = 31,860).

Institutional delivery was defined as delivery in a medical facility, dichotomized as yes or no. Pregnant women who deliver in a government medical facility or in a medical facility accredited by the Government of India are eligible to receive financial incentives under the JSY. Under this scheme, financial incentives are directly transferred to the bank account of the beneficiary. Hence, there is a possibility of endogeneity when examining the association between bank account ownership and institutional delivery. In presence of endogeneity, the regression coefficients are biased (Greene, 2012; Kennedy, 2003). To avoid this problem, we restricted the analysis of institutional delivery to most recent singleton births in the past five years for which mothers did not receive financial incentive under JSY (n = 33,912).

The primary independent variable included in the analysis is the women's ownership of a bank account that they themselves can use, dichotomized as yes or no. The question on women's ownership of a bank account was canvassed in a sub-sample of 15% of the randomly selected households. Women in these sub-sample of households were asked:

Do you have a bank or savings account that you yourself use? (Yes/No).

Women who reported 'Yes' were coded as having a bank account and others as not having a bank account.

Additional covariates included in the analysis are women's/mother's age, women's/mother's schooling, women's/mother's age at marriage, mother's parity, experience of child loss, women's/mother's work status, religion, wealth quintiles, urban-rural residence, and geographic region of residence.^1^ Experience of pregnancy complications was additionally included in the ANC analysis. Similarly, ANC was included as a covariate in the institutional delivery analysis. The wealth quintiles are already estimated and given in the NFHS-4 dataset. The wealth quintiles in NFHS-4 are principal component analysis-derived index of household assets and amenities.

### 2.3 Statistical methods

We used multivariable binary logistic regression models to examine association of ownership of a bank account with birth interval and institutional delivery. We used multivariable multinomial logistic regression models to examine association of ownership of a bank account with current use of contraceptives and availing ANC visits. We estimated two multivariable multinomial logistic regression models for availing ANC visits. The first model included all most recent singleton births in five years preceding NFHS-4. To overcome the issue of endogeneity, we included only those births in the second model for whom mothers did not receive any financial incentive under JSY for delivering their babies in government medical facilities. Finally, we estimated the afore-mentioned models separately for 21 bigger states of India to examine if the associations between women's ownership of a bank account and selected outcomes vary by state.

A key concern while analyzing the association between women's ownership of a bank account and selected reproductive and maternal health services utilization and behavior is that women owning a bank account may be selective on a whole set of characteristics that might influence the outcomes considered. We used propensity score matching (PSM) to account for this potential selectivity in our sample. PSM is a statistical technique that estimates the effect of a treatment or intervention by adjusting for covariates that predict receiving the treatment or intervention (Rosenbaum & Rubin, 1983). For computing the average treatment effect (i.e., the effect of ownership of a bank account), a counterfactual model is estimated. The counterfactual is the potential outcome that we would have obtained in case the women do not own a bank account. With the help of the counterfactual model, the average treatment effect on the treated (ATT) is estimated as:

ATT=E(Y1/D=1)−E(Y0/D=1),

where E(Y_1_/D = 1) gives the utilization of health services for women who own a bank account and E(Y_0_/D = 1) is the expected outcome if women owning a bank account were not to own it.

Similarly, the average treatment effect on the untreated (ATU) is defined mathematically as:

ATU=E(Y1/D=0)−E(Y0/D=0),

where E(Y_1_/D = 0) is the expected outcome if women who do not own a bank account were to own a bank account and E(Y_0_/D = 0) is the outcome for women who do not own a bank account.

The average treatment effect (ATE) is the difference between the expected outcome for women who own a bank account and women who do not own a bank account. The details of PSM can be obtained elsewhere (Caliendo & Kopeinig, 2005; Heckman, Lalonde, & Smith, 1999; Rosenbaum & Rubin, 1983; Sianesi, 2004; Singh, Upadhyay, Singh, & Kumar, 2017). Current use of contraceptives was coded into binary variable having two categories (no, yes) for the PSM analysis. Likewise availing ANC visits was coded into binary variable having two categories (did not avail ANC visit, availed ANC visit).

We weighted all analyses using NFHS-4 provided sampling weights to account for survey design.

## 3 Results

### 3.1 Sample characteristics

Table 1 shows the sample characteristics. Forty-one percent of currently married women age 15–49 reported not using any contraceptive method; 13% percent and 40% reported using modern spacing^2^ and modern limiting^3^ methods, respectively. About 7% of women reported using traditional^4^ methods. Among most recent higher order births, 27% had shorter term birth spacing, or <24 months between births, and an additional 32% had an interval of 24 months to <36 months between births. For most recent births in the past five years, 55% received 4 or more ANC visits; 30% received 1–3 ANC visits, and 15% did not receive ANC. For most recent births in the past five years, 75% of women reported an institutional delivery. Fifty-three percent of women age 15–49 in the current contraceptive use sample reported owning a bank account that they themselves use (Table 2). A little less than half of women in preceding birth interval (46%), availing ANC visits (49%), and institutional delivery (46%) samples reported owning a bank account, respectively.

**Table 1:** Sample description of dependent variables.

Variable	%	N (weighted)
Current contraceptive use		
Not using	40.9	33,286
Using modern spacing methods	12.5	10,187
Using modern limiting methods	40.0	32,585
Using traditional methods	6.7	5421
Preceding birth interval (less than 24 months)		
Less than 24 months	27.1	7119
Greater than or equal to 24 months	72.9	19,157
Preceding birth interval (less than 36 months)		
Less than 36 months	58.8	15,450
Greater than or equal to 36 months	41.2	10,826
Availing ANC visits		
No visits	15.3	4887
1 to 3 visits	29.8	9492
4 or more visits	54.9	17,481
Institutional delivery (among those births for which mothers did not receive incentive under JSY)		
No	25.3	8584
Yes	74.7	25,327

**Table 2:** Sample description (percent distribution), India, 2015-16.

Covariate/category	Current contraceptive use (N = 81,480)	Preceding birth interval (26,276)	Availing antenatal visits (N = 31,860)	Institutional delivery (N = 33,912)
**Owns a bank account**				
No	46.7 (38,044)	54.1 (14,205)	51.2 (16,319)	54.2 (18,365)
Yes	53.3 (43,436)	45.9 (12,071)	48.8 (15,541)	45.8 (15,547)
**Women's/mother's age**				
15-19	3.0 (2427)	0.6 (146)	3.4 (1080)	2.8 (943)
20-24	13.7 (11,185)	19.8 (5197)	30.9 (9834)	31.4 (10,642)
25-29	19.4 (15,828)	42.4 (11,131)	37.7 (12,002)	39.2 (13,293)
30-34	18.7 (15,203)	24.5 (6443)	18.7 (5971)	18.1 (6140)
35-39	17.1 (13,946)	9.4 (2477)	6.9 (2196)	6.4 (2163)
40-44	14.6 (11,921)	2.6 (682)	1.9 (605)	1.7 (568)
45-49	13.5 (10,970)	0.8 (200)	0.5 (172)	0.5 (163)
**Women's/mother's schooling**				
No schooling	32.1 (26,124)	36.8 (9674)	26.3 (8380)	28.1 (9519)
Up to primary	14.1 (11,515)	14.9 (3928)	12.9 (4118)	13.0 (4422)
Up to secondary	43.3 (35,243)	41.3 (10,839)	48.0 (15,276)	46.6 (15,812)
More than				
secondary	10.6 (8598)	7.0 (1835)	12.8 (4086)	12.3 (4159)
**Women's/mother's age at marriage**^a^				
<15	16.0 (12,539)	14.2 (3639)	10.4 (3235)	10.4 (3436)
15-17	29.3 (23,028)	32.2 (8239)	27.8 (8668)	28.2 (9351)
>=18	54.7 (42,993)	53.6 (13,724)	61.9 (19,329)	61.5 (20,393)
**Mother's parity**				
0	7.8 (6382)		–	
1	17.5 (14,287)	–	33.9 (10,798)	22.8 (7732)
2	34.3 (27,942)	41.9 (11,019)	34.6 (11,025)	39.3 (13,321)
3	20.4 (16,628)	29.2 (7679)	16.6 (5286)	19.9 (6740)
4 or more	19.9 (16,241)	28.8 (7578)	14.9 (4752)	18.1 (6119)
**Experienced child loss**				
No	87.5 (71,306)	80.7 (21,201)	88.4 (28,163)	85.6 (29,016)
Yes	12.5 (10,174)	19.3 (5075)	11.6 (3697)	14.4 (4896)
**Women's/mother's work status**				
Not working	75.2 (61,256)	81.0 (21,291)	83.0 (26,452)	83.6 (28,336)
Working	24.8 (20,224)	19.0 (4985)	17.0 (5408)	16.4 (5576)
**Religion**				
Hindu	81.5 (66,452)	77.5 (20,376)	78.9 (25,130)	77.6 (26,318)
Muslim	13.3 (10,805)	18.1 (4750)	16.3 (5183)	17.3 (5868)
Other	5.2 (4223)	4.4 (1150)	4.9 (1547)	5.1 (1726)
**Wealth quintiles**				
Poorest	16.2 (13,167)	28.9 (7606)	22.1 (7029)	22.8 (7726)
Poorer	19.0 (15,445)	23.0 (6050)	20.8 (6611)	20.3 (6868)
Middle	20.7 (16,896)	19.6 (5155)	20.4 (6483)	20.1 (6805)
Richer	21.6 (17,610)	15.9 (4174)	18.9 (6028)	19.0 (6443)
Richest	22.5 (18,362)	12.5 (3291)	17.9 (5709)	17.9 (6070)
**Urban-rural residence**				
Urban	34.9 (28,451)	26.3 (6902)	30.8 (9827)	31.8 (10,790)
Rural	65.1 (53,029)	73.7 (19,374)	69.2 (22,033)	68.2 (23,122)
**Region of residence**				
South	25.1 (20,414)	16.9 (4441)	20.2 (6441)	21.2 (7202)
North	13.2 (10,797)	13.4 (3515)	13.0 (4153)	12.8 (4329)
Central	20.4 (16,628)	28.0 (7356)	23.8 (7596)	23.2 (7875)
East	21.5 (17,516)	24.9 (6554)	24.2 (7708)	22.2 (7540)
Northeast	3.2 (2625)	3.2 (852)	3.7 (1162)	2.9 (977)
West	16.6 (13,500)	13.5 (3558)	15.1 (4800)	17.7 (5989)

Notes: Weighted Ns are given in the parentheses

a Does not add to N due to inconsistent/do not know cases.

### 3.2 Bivariate results

**Fig. 1:**
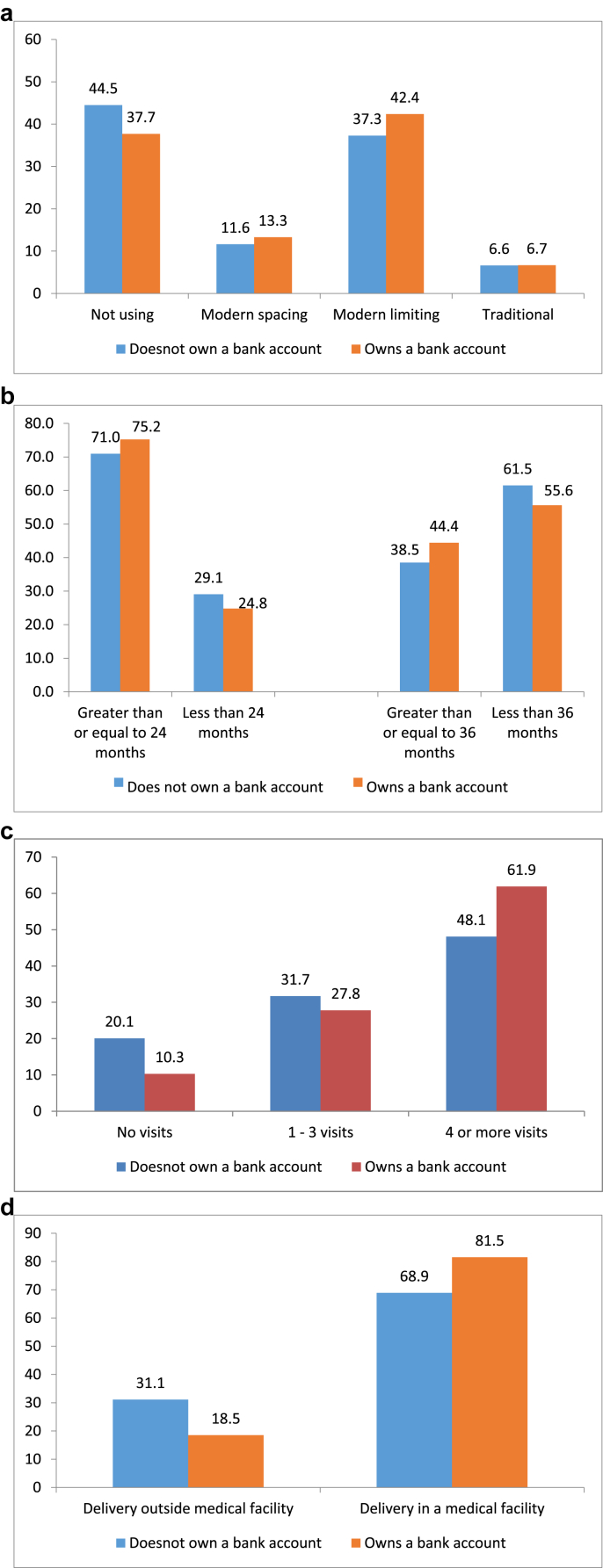
a) Percent of women currently using contraceptives by ownership of a bank account, India, 2015-16. (b): Among those with at least two children, median preceding birth interval by ownership of a bank account, India, 2015-16. (c): Percent of women availing ANC visits for their most recent birth in five years preceding the survey by ownership of a bank account, India, 2015-16. (d): Percent of women delivering in a medical facility in five years preceding the survey by ownership of a bank account, India, 2015-16.

Fig. 1a–d show bivariate association between women's ownership of a bank account and the selected reproductive and maternal health services utilization and behavior. More women owning a bank account reported using modern spacing and modern limiting methods compared with women not owning a bank account. Likewise, women owning a bank account reported larger preceding birth intervals compared with women not owning a bank account. Twenty-five percent and 56% of women owning a bank account reported a preceding birth interval of less than 24- and 36- months, respectively. In comparison, 29% and 62% of women who did not own a bank account reported a preceding birth interval of less than 24- and 36- months, respectively. Women owning a bank account were also more likely to avail 4 or more ANC visits. For example, 62% of women owning a bank account availed 4 or more ANC visits for their most recent birth compared with only 48% of women not owning a bank account. Institutional delivery was also higher among women owning a bank account compared with women not owning an account.

### 3.3 Results from multivariable logistic regression analyses

Women's ownership of a bank account was associated with higher use of modern spacing methods and traditional methods (Table 3). Women owning a bank account were 1.20 times as likely as women not owning a bank account to use a modern spacing method. Likewise, women owning a bank account were 1.15 times as likely as their counterparts to use traditional methods. Women's ownership of a bank account was associated with larger preceding birth intervals in multivariate analysis. Women owning a bank account were only 0.82 times and 0.85 times as likely as their counterparts to have preceding birth interval of less than 24- or 36- months (Table 4).

**Table 3:** Results of multinomial logistic regression (RRR) assessing the association between ownership of a bank account and current use of contraception^a^ by women, India, 2015-16.

Covariate/category	Modern spacing	Modern limiting	Traditional
**Owns a bank account**			
No (r)			
Yes	1.20 (1.14,1.26)*	0.99 (0.95,1.03)	1.15 (1.08,1.23)*
**Women's schooling**			
No schooling (r)			
Up to primary	1.72 (1.57,1.88)*	1.24 (1.16,1.31)*	1.36 (1.22,1.50)*
Up to secondary	1.81 (1.68,1.96)*	0.97 (0.92,1.03)*	1.48 (1.36,1.62)*
More than secondary	2.24 (2.02,2.49)*	0.65 (0.59,0.71)*	1.30 (1.14,1.49)*
**Women's age at marriage**			
>=18 (r)			
<15	0.65 (0.60,0.71)*	1.30 (1.23,1.37)*	0.77 (0.70,0.85)*
15-17	0.97 (0.91,1.03)	1.49 (1.42,1.56)*	0.97 (0.90,1.05)
**Experienced child loss**			
No (r)			
Yes	0.93 (0.85,1.02)	0.72 (0.68,0.76)*	0.95 (0.86,1.05)
**Women's work status**			
Not working			
Working	1.22 (1.15,1.30)*	1.39 (1.33,1.45)*	1.22 (1.13,1.32)*
Religion			
Hindu (r)			
Muslim	1.48 (1.39,1.58)*	0.36 (0.34,0.39)*	1.05 (0.96,1.14)
Other	1.06 (0.95,1.18)	0.93 (0.85,1.01)	0.96 (0.83,1.11)
**Wealth quintiles**			
Poorest (r)			
Poorer	1.60 (1.46,1.75)*	1.33 (1.25,1.41)*	1.40 (1.27,1.55)*
Middle	1.82 (1.66,2.00)*	1.44 (1.34,1.54)*	1.56 (1.40,1.74)*
Richer	2.08 (1.88,2.30)*	1.59 (1.48,1.72)*	1.81 (1.62,2.04)*
Richest	2.73 (2.44,3.04)*	1.40 (1.28,1.52)*	1.76 (1.54,2.01)*
**Urban-rural residence**			
Urban (r)			
Rural	0.84 (0.79,0.89)*	1.10 (1.05,1.16)*	0.91 (0.84,0.98)*

Notes:*p < 0.05.

‘r’ indicates reference category values in the parentheses are 95% confidence interval.

Results are adjusted for age and parity of women, and region of residence.

a The outcome reference group is women who are currently not using any contraceptive.

**Table 4:** Results of binary multivariable logistic regression assessing the association between ownership of a bank account and having preceding birth intervals less than 24 months or 36 months, India, 2015-16.

Covariate/category	Model 1 (<24 months)	Model 2 (<36 months)
**Owns a bank account**		
No (r)		
Yes	0.82 (0.76,0.89)*	0.85 (0.79,0.92)*
**Mother's schooling**		
No schooling (r)		
Up to primary	0.96 (0.86,1.08)	0.91 (0.81,1.02)
Up to secondary	0.96 (0.86,1.06)	0.81 (0.74,0.90)*
More than secondary	1.17 (0.94,1.46)	0.96 (0.79,1.15)
**Mother's age at marriage**		
>=18 (r)		
<15	0.36 (0.32,0.41)*	0.31 (0.27,0.35)*
15-17	0.54 (0.49,0.59)*	0.48 (0.44,0.52)*
**Mother's parity**		
2 (r)		
3	1.36 (1.23,1.51)*	1.66 (1.51,1.82)*
4 or more	2.21 (1.95,2.49)*	3.48 (3.10,3.92)*
**Experienced child loss**		
No (r)		
Yes	1.55 (1.41,1.71)*	1.17 (1.07,1.29)*
**Mother's work status**		
Not working (r)		
Working	0.99 (0.89,1.09)	1.03 (0.93,1.13)
**Religion**		
Hindu (r)		
Muslim	0.98 (0.89,1.09)	0.85 (0.77,0.94)*
Other	0.96 (0.78,1.18)	0.89 (0.73,1.09)
**Wealth quintiles**		
Poorest (r)		
Poorer	1.11 (1.00,1.23)	0.98 (0.88,1.08)
Middle	1.08 (0.96,1.22)	0.87 (0.77,0.97)*
Richer	1.04 (0.90,1.20)	0.87 (0.76,0.99)*
Richest	0.75 (0.63,0.91)*	0.60 (0.51,0.71)*
**Urban-rural residence**		
Urban (r)		
Rural	1.15 (1.03,1.29)*	1.16 (1.05,1.29)*
**Region of residence**		
South (r)		
North	0.99 (0.86,1.14)	1.06 (0.92,1.21)
Central	0.90 (0.79,1.03)	0.92 (0.81,1.05)
East	0.72 (0.62,0.83)*	0.66 (0.57,0.76)*
Northeast	0.53 (0.44,0.64)*	0.43 (0.37,0.51)*
West	0.75 (0.62,0.90)*	0.87 (0.73,1.03)

Notes:*p < 0.05, values in the parentheses are 95% confidence interval.

‘r’ indicates reference category.

Results are adjusted for mother's age.

The association between ownership of a bank account and having preceding birth intervals less than 24 months or 36 months was significant even after adjusting for current use of contraception.

Table 5 shows the results of multivariable multinomial logistic regression assessing the association between mother's ownership of a bank account and availing ANC visits. Mothers owning a bank account were 1.57 times as likely as mothers not owning a bank account to avail the recommended 4 or more ANC visits. Likewise, mothers owning a bank account were 1.41 times as likely as mothers not owning a bank account to avail 1–3 ANC visits. The regression model run on only those recent births for whom mothers did not receive incentive under JSY also yielded similar results. For example, mothers owning a bank account were 1.17 and 1.39 times as likely as mothers not owning a bank account to have availed 1–3 and 4 or more ANC visits. Unlike the other outcomes, mother's ownership of a bank account was not associated with institutional delivery in the multivariate analysis (Table 6).

**Table 5:** Results of multinomial logistic regression (RRR) assessing the association between ownership of a bank account and availing four or more ANC visits,^a^ India, 2015-16.

Covariate/category	All most recent births		Most recent birth for whom mother did not receive incentive under JSY	
	1 to 3 ANC visits	4 or more visits	1 to 3 ANC visits	4 or more visits
**Owns a bank account**				
No (r)				
Yes	1.41 (1.31,1.53)*	1.57 (1.46,1.70)*	1.17 (1.02,1.35)*	1.39 (1.22,1.59)*
**Mother's schooling**				
No schooling (r)				
Up to primary	1.41 (1.27,1.58)*	1.87 (1.67,2.09)*	1.39 (1.11,1.73)*	1.66 (1.34,2.05)*
Up to secondary	1.49 (1.35,1.64)*	2.18 (1.98,2.40)*	1.43 (1.20,1.72)*	1.71 (1.44,2.04)*
More than secondary	1.46 (1.20,1.78)*	2.60 (2.15,3.14)*	1.39 (1.03,1.89)*	2.22 (1.68,2.95)*
**Mother's age at marriage**				
>=18 (r)				
<15	0.90 (0.81,1.01)	0.77 (0.68,0.86)*	1.03 (0.82,1.30)	0.94 (0.75,1.17)
15-17	0.92 (0.85,1.01)	0.93 (0.85,1.01)	0.96 (0.81,1.13)	0.97 (0.83,1.13)
**Mother's parity**				
1 (r)				
2	0.85 (0.76,0.94)*	0.75 (0.67,0.83)*	0.75 (0.63,0.89)*	0.73 (0.62,0.86)*
3	0.73 (0.65,0.83)*	0.51 (0.45,0.57)*	0.80 (0.64,1.01)	0.71 (0.57,0.88)*
4 or more	0.63 (0.54,0.73)*	0.32 (0.28,0.37)*	0.74 (0.56,0.98)*	0.52 (0.40,0.68)*
**Experienced child loss**				
No (r)				
Yes	1.06 (0.96,1.18)	1.07 (0.96,1.19)	1.06 (0.87,1.31)	0.93 (0.76,1.14)
**Experienced pregnancy complication**				
No (r)				
Yes	1.06 (0.99,1.14)	1.16 (1.08,1.25)*	0.98 (0.86,1.12)	1.04 (0.92,1.18)
**Mother's work status**				
Not working (r)				
Working	1.07 (0.97,1.18)	1.08 (0.98,1.18)	1.42 (1.16,1.73)	1.37 (1.13,1.66)*
**Religion**				
Hindu (r)				
Muslim	0.97 (0.88,1.07)	1.15 (1.04,1.26)*	1.15 (0.95,1.39)	1.31 (1.10,1.57)*
Other	1.15 (0.93,1.43)	1.35 (1.10,1.66)*	1.54 (1.06,2.24)*	1.66 (1.17,2.37)*
**Wealth quintiles**				
Poorest (r)				
Poorer	1.35 (1.23,1.48)*	1.83 (1.65,2.01)*	1.53 (1.26,1.86)*	2.17 (1.79,2.63)*
Middle	1.48 (1.32,1.66)*	2.57 (2.29,2.88)*	1.53 (1.23,1.90)*	3.29 (2.67,4.04)*
Richer	1.81 (1.56,2.09)*	3.65 (3.16,4.21)*	2.17 (1.69,2.80)*	4.92 (3.87,6.25)*
Richest	2.04 (1.69,2.47)*	5.37 (4.47,6.45)*	2.24 (1.66,3.01)*	6.58 (4.96,8.72)*
**Urban-rural residence**				
Urban (r)				
Rural	1.06 (0.95,1.17)	0.95 (0.86,1.05)	1.10 (0.93,1.30)	1.01 (0.86,1.18)
**Region of residence**				
South (r)				
North	1.44 (1.22,1.71)*	0.45 (0.38,0.52)*	1.51 (1.17,1.96)*	0.53 (0.42,0.67)*
Central	1.23 (1.06,1.42)*	0.22 (0.19,0.26)*	1.23 (0.99,1.53)	0.27 (0.22,0.34)*
East	0.86 (0.74,1.00)	0.34 (0.30,0.39)*	0.82 (0.66,1.03)	0.45 (0.37,0.55)*
Northeast	2.09 (1.62,2.69)*	0.69 (0.54,0.89)*	1.94 (1.14,3.31)*	0.70 (0.42,1.17)
West	0.89 (0.74,1.06)	0.79 (0.68,0.93)*	0.85 (0.68,1.07)	0.82 (0.66,1.00)

Notes: *p < 0.05, values in the parentheses are 95% confidence interval.

‘r’ indicates reference category.

Results are adjusted for mother's age.

a The outcome reference group is women who did not avail any antenatal visit.

**Table 6:** Results of multivariable binary logistic regression assessing the association between ownership of a bank account and institutional delivery, India, 2015-16.

Covariate/category	Institutional delivery
Owns a bank account	
No (r)	
Yes	1.05 (0.94,1.17)
Mother's age	
15-19 (r)	
20–24	0.89 (0.64,1.22)
25–29	1.03 (0.74,1.45)
30–34	1.20 (0.84,1.71)
35–39	1.19 (0.81,1.74)
40–44	0.85 (0.54,1.33)
45–49	0.56 (0.29,1.07)
Mother's schooling	
No schooling (r)	
Up to primary	1.04 (0.89,1.22)
Up to secondary	1.57 (1.37,1.80)*
More than secondary	3.12 (2.35,4.14)*
Mother's age at marriage	
>=18 (r)	
<15	0.93 (0.79,1.09)
15–17	0.89 (0.78,1.00)
Mother's parity	
1 (r)	
2	0.48 (0.41,0.56)*
3	0.33 (0.27,0.39)*
4 or more	0.28 (0.23,0.35)*
Experienced child loss	
No (r)	
Yes	1.31 (1.13,1.52)*
Antenatal visits	
No visits (r)	
1-3 visits	1.66 (1.45,1.89)*
4 or more visits	2.99 (2.59,3.46)*
Mother's work status	
Not working (r)	
Working	0.62 (0.55,0.71)*
Religion	
Hindu (r)	
Muslim	0.64 (0.56,0.73)*
Other	0.91 (0.71,1.15)
Wealth quintiles	
Poorest (r)	
Poorer	1.69 (1.47,1.94)*
Middle	2.45 (2.09,2.87)*
Richer	3.27 (2.67,4.02)*
Richest	6.37 (4.90,8.28)*
Urban-rural residence	
Urban (r)	
Rural	0.87 (0.75,1.00)
Region of residence	
South (r)	
North	0.17 (0.13,0.22)*
Central	0.10 (0.08,0.13)*
East	0.13 (0.10,0.17)*
Northeast	0.07 (0.06,0.10)*
West	0.43 (0.32,0.59)*

Notes: *p < 0.05, Values in the parentheses are 95% confidence interval.

‘r’ indicates reference category.

Women's schooling, women's working status, wealth quintiles, and urban-rural residence were associated with current use of contraceptives in the multivariate analysis. When it comes to preceding birth intervals, age at marriage, parity, experience of child loss, and urban-rural residence showed significant association. While mother's schooling, wealth quintiles, and experience of child loss were positively associated with availing ANC visits and institutional delivery, parity was negatively associated. The two outcomes also varied considerably by region of residence. Availing ANC visits was also positively associated with institutional delivery, while working status of mother was negatively associated.

### 3.4 State-specific results from multivariable logistic regression analyses

State-specific results are shown in Appendices A1-A4. Observed associations are strongest in states where the reproductive and maternal health services utilization is far below the national average. For example, women's ownership of a bank account was associated with higher use of modern spacing methods in Jammu & Kashmir, Rajasthan, Uttar Pradesh, Madhya Pradesh, and Bihar. Likewise, women's ownership of a bank account was associated with a higher use of modern limiting methods in Jammu & Kashmir, Himachal Pradesh, Haryana, Rajasthan, Uttar Pradesh, Chhattisgarh, and Bihar. Women's ownership of a bank account was negatively associated with shorter birth intervals in Rajasthan, Uttar Pradesh, and Assam. The association between mother's ownership of a bank account and availing the recommended 4 or more ANC visits clearly stood out in Jammu & Kashmir, Rajasthan, Uttar Pradesh, Madhya Pradesh, Bihar, Jharkhand, Odisha, and Assam. Although, mother's ownership of a bank account was not associated with institutional delivery at the national level, but was associated with institutional delivery in Jammu & Kashmir, Rajasthan, and Karnataka.

### 3.5 Results from propensity score matching analysis

A concern while analyzing the association between women's ownership of a bank account and selected outcomes is the selectivity bias in bank account ownership status. The characteristics of women/mothers who report owning a bank account suggest that these women/mothers are selective on a number of characteristics associated with utilization of the selected health services and behavior. Women/mothers owning a bank account were particularly selective on schooling, age at marriage, wealth quintiles, urban-rural residence, and region of residence (Appendix A5). We explored the effect of this selectivity bias using PSM. The results of propensity score matching analyses are shown in Table 7. The unmatched sample estimates for current use of contraceptives shows that the difference in the current use of contraception by those who own a bank account and those who do not own is 0.08 (or 8%). This indicates that women who own a bank account are more likely to use contraceptives compared to those who do not own a bank account. The estimated ATT values in treated and control groups are 0.502 and 0.470 respectively thus indicating that the current use of contraceptives increased by 3 percentage points because of ownership of a bank account. ATU results indicate that among those women who do not own a bank account if were to own a bank account, the current use of contraceptives is likely to increase by 3 percentage points.

**Table 7:** Results of matching estimates showing the effect of having a bank account on utilization of the three selected health services by women, India, 2015-16.

Having a bank account versus not having a bank account	Treated	Controls	Differences	S.E.	p > z	95% CI
**Current contraceptive use**						
Unmatched	0.502	0.426	0.075	0.005		
ATT	0.502	0.470	0.032	0.012	0.000	(0.008,0.056)
ATU	0.426	0.456	0.030			
ATE			0.031			
**Preceding birth interval less than 24 months**						
Unmatched	0.242	0.282	−0.040	0.005		
ATT	0.243	0.266	−0.024	0.012	0.044	(-0.056, −0.001)
ATU	0.282	0.226	−0.056			
ATE			−0.041			
**Preceding birth interval less than 36 months**						
Unmatched	0.545	0.615	−0.070	0.006		
ATT	0.546	0.569	−0.022	0.010	0.000	(-0.042, −0.002)
ATU	0.615	0.587	−0.028			
ATE			−0.025			
**Availing ANC visit**						
Unmatched	0.893	0.784	0.109	0.004		
ATT	0.893	0.855	0.038	0.010	0.000	(0.019,0.056)
ATU	0.784	0.846	0.062			
ATE			0.050			

Note: The balancing property was satisfied at p < 0.005.

Similarly, the unmatched sample estimates for preceding birth interval less than 24 months shows that women owning a bank account were less likely to have preceding birth interval shorter than 24 months compared with women not owning a bank account. The estimated ATT values in treated and control groups are 0.243 and 0.266. This indicates that the prevalence of preceding birth interval less than 24 months decreased by 2 percentage points because of ownership of a bank account. ATU results indicate that among those women who do not own a bank account if were to own a bank account, the prevalence of preceding birth interval less than 24 months is likely to decrease by 6 percentage points.

The propensity score results for preceding birth interval less than 36 months and availing ANC visits suggest that women's/mothers' ownership of a bank account is indeed associated with larger preceding birth interval and availing ANC visits even after accounting for sample selectivity bias.

## 4 Discussion

The evidence from the study suggest that women's ownership of a bank account is associated with modern contraceptive use, greater birth spacing, and receipt of ANC, three of the four outcomes we analyzed. The associations were significant even after adjusting for relevant socio-economic, demographic, and residence related characteristics. The propensity score results indeed support the findings obtained from the multivariable regression models. These findings build upon previous research documenting the value of women's financial inclusion measures as a means of support to women's health (Hamad & Fernald, 2015; Kim et al., 2017; Mohindra et al., 2008; Orton et al., 2016; Raj et al., 2018; Schuler & Hashemi, 1994; Steele et al., 1998), and extend these findings by documenting more specifically the value of women's bank account ownership for reproductive and maternal health services utilization and behavior.

Although the results of our study are reassuring, identifying the specific channels through which women's ownership of a bank account may influence these outcomes is unclear. We understand that women's ownership of a bank account may give them more autonomy and control over important decisions affecting them and their children, as well as the mobility and self-efficacy to act upon their decisions. The NFHS-4 suggests that women who own a bank account were indeed more likely to a) have ability to go for medical care for themselves, b) be allowed to go to a health facility alone, c) be allowed to go to a place outside community alone, and d) be allowed to keep some money aside that they themselves can decide how to use compared with women not owning a bank account (Results not shown). Interestingly, women who own a bank account were more likely to decide how to spend husband's earning compared with women who do not own a bank account. Additional evidence from these data indeed suggests that women who owned a bank account were more likely than women who do not own a bank account to have heard family planning messages on radio or television or read in newspaper/magazine in last few months (results not shown). Women age 15–49 who reported not using any contraceptive in NFHS-4 were further asked if they know of a place where they can obtain a method of family planning. Sixty-nine percent of women owning a bank account as opposed to only 49% of women not owning a bank account reported that they knew a place where they can obtain a method of family planning. These additional analyses suggest that bank account ownership is in fact related to women's empowerment and may also contribute to increasing self-confidence and ability of women to plan better for their future. Women's financial inclusion in the form of ownership of a bank account might also provide women with opportunity to mix and interact with women from other communities, thereby increasing their knowledge about issues that are of interest to women (Desai and Tarozzi, 2011). Research in similar settings has shown that women's financial inclusion might improve the woman's bargaining power and control over important decisions including finances and health (Desai and Tarozzi, 2011; Hennink & McFarland, 2013; Schuler & Hashemi, 1994). Studies have also shown that without the help of banks, savings are at greater risk and grow more slowly (Mullainathan & Shafir, 2010). Such a situation might not allow women to plan better for their future. Further research, including qualitative research will be important in offering more insight into these issues.

A key finding that deserves mention is the association between women's ownership of a bank account and the selected outcomes in the poor performing states of India. Women owning a bank account in Jammu & Kashmir, Rajasthan, Uttar Pradesh, Madhya Pradesh, and Bihar were more likely to use modern spacing methods compared to women who did own a bank account. Women owning a bank account in Jammu & Kashmir, Himachal Pradesh, Haryana, Rajasthan, Uttar Pradesh, Chhattisgarh, and Bihar were also more likely to use modern limiting methods. Likewise, use of 4 or more ANC visits was higher in women owning a bank account in Jammu & Kashmir, Rajasthan, Uttar Pradesh, Madhya Pradesh, Bihar, Jharkhand, Odisha, and Assam compared with women not owning a bank account. Although ownership of a bank account was not statistically associated with delivery in a medical facility at the national level, it was statistically associated with delivery in a medical facility in Jammu & Kashmir, Rajasthan, and Karnataka. Clearly, a majority of these states have much lower utilization of the selected health services compared to the other more developed states. For example, the current contraceptive use is only 24% in Bihar whereas the national average is 54%. Uttar Pradesh and Madhya Pradesh are also below the national average. When it comes to 4 or more ANC visits, the national average is 51%. In comparison, only 14% of women in Bihar availed 4 or more ANC visits for their most recent birth in 5 years preceding NFHS-4. The comparable percentages for Uttar Pradesh, Jharkhand, Madhya Pradesh, Rajasthan, and Assam are 26%, 30%, 36%, 39%, and 46% respectively. Even Odisha has much lower coverage of 4 or more ANC visits compared to the more developed south Indian states. There is ample scope for improvement in selected reproductive and maternal health services utilization and behavior in these poor performing states and providing access to a bank account is one of the options. Improving women's schooling and economic status of the households in these states is equally important because even women owning a bank account in these states perform poorer in terms of the reproductive and maternal health services utilization and behavior compared with women owning a bank account in better performing states like Maharashtra, Kerala, Tamil Nadu, etc. For example, only 39% of women owning a bank account in Bihar reported using any contraceptive method compared with 59% of women owning a bank account in Tamil Nadu. Likewise, only 29% of mothers owning a bank account in Bihar availed 4 or more ANC visits. In comparison, 83% and 75% of women owning a bank account in Tamil Nadu and Maharashtra availed 4 or more ANC visits, respectively.

Our findings lend support to the Government of India's recent PMJDY initiative as both a financial inclusion scheme and a support for women's health care utilization. This indirect benefit for women's health care use is likely to be greater in poor performing, less developed states of India. Recent statistics suggest that over 330 million bank accounts were opened under PMJDY (Government of India, 2014). Of these, 180 million accounts were opened in rural or semi-urban areas. Further, as noted above, there has been a dramatic increase in bank account ownership among women, even since these NFHS-4 data were collected in 2015–16; where NFHS-4 findings indicate that 53% of women have a bank account, 2017 data reveal that 77% of females own a bank account (Demirgue-Kunt et al., 2017). While these improvements are notable, there remains a 6% gender gap in bank account ownership (Demirgue-Kunt et al., 2017). Strengthening PMJDY and pursuing it as a national mission might help in improving the coverage of selected health services in states where it is badly needed. Women's financial inclusion in the form of ownership of a bank account might also help in reducing disparities in reproductive and maternal health services utilization and behavior.

A key strength of our study is the use of a large-scale population based representative household survey data. NFHS-4 for the first time has provided us with a unique opportunity to examine association between women's ownership of a bank account and some selected outcomes. Even in such large-scale representative datasets, there is always a possibility that women who own a bank account may be selective on a whole set of characteristics that are associated with better outcomes. We used propensity score matching to address this selectivity in the sample. The propensity score matching analysis indeed confirmed the findings from the multivariable regression models. Thus, we believe that our results are robust to selectivity of women in the sample. The limitations of our study may also be noted. Endogeneity may be an issue while examining association between women's ownership of a bank account and higher utilization of delivery care. This is because women must have a bank account to receive the financial incentive for delivering in a medical facility. So, women who deliver in a medical facility are more likely to own a bank account. To address the issue of endogeneity, we restricted the analysis of institutional delivery to only those women who did not receive any financial incentive under JSY. Although we get away with a major chunk of the endogeneity issue, we cannot completely rule out the issue of endogeneity from our analysis. The issue of endogeneity might also apply to some extent to the analysis of ANC visits. Hence, we estimated two separate logistic regression models - one on all eligible births and second on births for whom mothers did not receive any incentive. Note that there is no separate financial incentive for availing recommended ANC visits under any of the schemes. This is also the case for contraceptive use. Second, we could not effectively examine the pathways through which women's ownership of a bank account might influence the selected outcomes, highlighting the need for more research on this issue.

## 5 Conclusions

This is perhaps the first study that provides estimates of the effect of women's financial inclusion on health benefits for women at a national scale. The strategy to provide a bank account to all in general and to the poor and the marginalized (including women) in particular seems to be effective. The strategy of providing a bank account is likely to pay higher dividends in states that are lacking behind in terms of reproductive and maternal health services utilization and behavior. However, more qualitative research is needed to identify the path ways through which women's financial inclusion might provide health benefits to women. We must also recognize that improving women's schooling and the economic status of households is important if India wants to achieve Sustainable Development Goal(SDG) 3: Improving Health and Well-Being, in conjunction with SDG 5: Achieving Gender Equality and Empowerment and SDG 10: Reducing inequalities.

## Conflicts of interest

None.

## Ethical statement

The paper uses National Family Health Survey 2015–16 (NFHS-4), a publicly available dataset with no identifiable information. The NFHS-4 can be freely downloaded from the DHS or the IIPS websites.

## Figures and Tables

**Table A1:** Results of multinomial logistic regression (RRR) assessing the association between ownership of a bank account and current use of contraception by women, selected states of India, 2015-16.

State	Modern spacing	Modern limiting	Traditional	Using a modern method
**North**				
Jammu & Kashmir	1.48 (1.23,1.77)*	1.64 (1.36,1.97)*	1.05 (085,1.31)	–
Himachal Pradesh	1.10 (0.81,1.49)	1.39 (1.07,1.81)*	1.57 (0.98,2.52)	–
Punjab	0.99 (0.74,1.31)	1.21 (0.90,1.62)	0.74 (0.53,1.05)	–
Haryana	1.11 (0.85,1.45)	1.41 (1.08,1.82)*	1.34 (0.85,2.12)	–
Uttarakhand	1.25 (0.94,1.66)	1.16 (0.87,1.53)	1.26 (0.74,2.13)	–
Rajasthan	1.33 (1.07,1.64)*	1.36 (1.16,1.60)*	1.18 (0.91,1.54)	–
**Central**				
Uttar Pradesh	1.26 (1.11,1.43)*	1.30 (1.15,1.47)*	1.10 (0.97,1.25)	–
Madhya Pradesh	1.38 (1.13,1.69)*	1.03 (0.91,1.17)	1.81 (1.26,2.61)*	–
Chattisgarh	1.23 (0.90,1.68)	1.26 (1.03,1.54)*	1.28 (0.85,1.93)	–
**East**				
Bihar	2.48 (1.78,3.45)*	1.40 (1.21,1.63)*	2.32 (1.31,4.11)*	–
Jharkhand	1.21 (0.88,1.66)	1.14 (0.95,1.36)	0.83 (0.55,1.25)	–
Odisha	0.97 (0.79,1.18)	0.94 (0.78,1.13)	1.17 (0.94,1.46)	–
West Bengal	1.11 (0.84,1.46)	1.11 (0.84,1.47)	0.72 (0.53,0.99)*	–
**Northeast**				
Assam	1.14 (0.94,1.38)	0.94 (0.70,1.26)	1.18 (0.95,1.48)	–
West				
Gujarat	1.00 (0.78,1.28)	0.99 (0.84,1.17)	1.27 (0.91,1.79)	–
Maharashtra	1.29 (0.99,1.69)	1.00 (0.81,1.24)	0.70 (0.45,1.10)	–
**South**^a^				
Andhra Pradesh	–	–		1.66 (1.13,2.42)*
Telangana	–	–		0.98 (0.65,1.47)
Karnataka	–	–		1.06 (0.77,1.47)
Kerala	–	–		0.88 (0.66,1.17)
Tamil Nadu	–	–		1.26 (1.02,1.56)*

Notes: *p < 0.05, values in the parentheses are 95% confidence intervals.

a Since, the frequency for modern spacing was small in Andhra Pradesh, Telangana, Karnataka, Kerala, and Tamil Nadu, the modern spacing and modern limiting were put together in the regression model.

**Table A2:** Results of binary multivariable logistic regression assessing the association between ownership of a bank account and having preceding birth intervals less than 24 months or 36 months, selected states of India, 2015-16.

State	Preceding birth interval less than 24 months	Preceding birth interval less than 36 months
**North**		
Jammu & Kashmir	1.25 (0.89,1.74)	1.04 (0.79,1.38)
Himachal Pradesh	0.64 (0.36,1.13)	1.19 (0.71,2.00)
Punjab	1.08 (0.61,1.92)	1.15 (0.64,2.05)
Haryana	0.99 (0.63,1.57)	1.05 (0.66,1.67)
Uttarakhand	0.72 (0.43,1.21)	0.68 (0.42,1.12)
Rajasthan	0.71 (0.55,0.91)*	0.89 (0.69,1.14)
**Central**		
Uttar Pradesh	0.79 (0.68,0.92)*	0.83 (0.72,0.96)*
Madhya Pradesh	1.04 (0.83,1.28)	0.88 (0.70,1.10)
Chattisgarh	1.22 (0.81,1.83)	0.97 (0.68,1.39)
**East**		
Bihar	0.84 (0.68,1.04)	0.82 (0.67,1.02)
Jharkhand	0.82 (0.59,1.13)	0.78 (0.59,1.02)
Odisha	0.90 (0.57,1.40)	1.27 (0.90,1.80)
West Bengal	0.97 (0.47,1.99)	1.05 (0.60,1.83)
**Northeast**		
Assam	0.59 (0.34,1.02)	0.54 (0.37,0.79)*
West		
Gujarat	0.86 (0.57,1.28)	1.04 (0.72,1.49)
Maharashtra	0.73 (0.44,1.21)	1.11 (0.70,1.75)
**South**		
Andhra Pradesh	1.27 (0.69,2.34)	1.02 (0.49,2.13)
Telangana	0.87 (0.42,1.84)	0.95 (0.44,2.09)
Karnataka	0.64 (0.40,1.03)	0.85 (0.55,1.32)
Kerala^a^		
Tamil Nadu	0.74 (0.48,1.17)	0.78 (0.50,1.22)

Notes: *p < 0.05, values in the parentheses are 95% confidence intervals.

a Multinomial logistic regression model did not converge in Kerala.

**Table A3:** Results of multinomial logistic regression (RRR) assessing the association between ownership of a bank account and availing four or more antenatal visits, selected states of India, 2015-16.

State	1 to 3 antenatal visits	4 or more antenatal visits	Availing antenatal visits
**North**			
Jammu & Kashmir	1.40 (0.84,2.32)	2.28 (1.52,3.43)*	
Himachal Pradesh^a^			
Punjab^c^	–	–	1.00 (0.64,1.57)
Haryana	0.92 (0.53,1.59)	1.70 (1.00,2.88)	
Uttarakhand	1.08 (0.65,1.77)	0.87 (0.50,1.50)	
Rajasthan	2.01 (1.47,2.73)*	2.83 (2.03,3.95)*	
**Central**			
Uttar Pradesh	1.45 (1.25,1.69)*	1.46 (1.22,1.76)*	
Madhya Pradesh	1.81 (1.41,2.32)*	1.86 (1.44,2.42)*	
Chattisgarh^c^	–	–	1.02 (0.74,1.41)
**East**			
Bihar	1.23 (0.99,1.52)	1.33 (1.00,1.77)*	
Jharkhand	1.57 (1.16,2.14)*	1.73 (1.23,2.44)*	
Odisha	2.28 (1.32,3.96)*	2.81 (1.65,4.78)*	
West Bengal	1.35 (0.61,3.00)	1.05 (0.53,2.12)	
**Northeast**			
Assam	1.72 (0.99,2.98)	2.65 (1.53,4.61)*	
**West**			
Gujarat	0.84 (0.53,1.35)	1.31 (0.90,1.92)	
Maharashtra	1.04 (0.56,1.95)	0.84 (0.47,1.49)	
**South**			
Andhra Pradesh^c^	–	–	1.12 (0.61,2.05)
Telangana^c^	–	–	0.58 (0.29,1.17)
Karnataka	0.79 (0.43,1.48)	0.96 (0.55,1.66)	
Kerala^b^			
Tamil Nadu	1.10 (0.59,2.07)	1.54 (0.94,2.51)	

Notes: *p < 0.05, values in the parentheses are 95% confidence intervals.

a Multinomial logistic regression model did not converge in Himachal Pradesh.

b Estimates could not be generated for Kerala due to small cell frequencies.

c Since, the frequency for no antenatal visits was small in Chattisgarh, Punjab, Andhra Pradesh, and Telangana, 1 to 3 antenatal visits and 4 or more antenatal visits were put together in the regression model.

**Table A4:** Results of multivariable binary logistic regression assessing the association between ownership of a bank account and delivery in medical institutions, selected states of India, 2015-16.

State	Delivery in a medical institution
**North**	
Jammu & Kashmir	1.50 (1.01,2.24)*
Himachal Pradesh	1.40 (0.83,2.36)
Punjab	1.39 (0.61,3.13)
Haryana	0.94 (0.50,1.76)
Uttarakhand	0.79 (0.43,1.45)
Rajasthan	1.48 (1.01,2.19)*
**Central**	
Uttar Pradesh	0.92 (0.76,1.11)
Madhya Pradesh	1.01 (0.69,1.50)
Chattisgarh	1.55 (0.96,2.51)
**East**	
Bihar	0.88 (0.64,1.23)
Jharkhand	1.07 (0.75,1.53)
Odisha	0.98 (0.56,1.69)
West Bengal	1.01 (0.57,1.82)
**Northeast**	
Assam	1.23 (0.72,2.08)
**West**	
Gujarat	1.24 (0.78,2.00)
Maharashtra	1.06 (0.54,2.07)
**South**	
Andhra Pradesh^a^	
Telangana^a^	
Karnataka	2.76 (1.13,6.73)*
Kerala^a^	
Tamil Nadu^a^	

Notes: *p < 0.05, values in the parentheses are 95% confidence intervals.

a Estimates could not be generated due to small cell frequencies.

**Table A5:** Characteristics of women who do not own and who own a bank account, India, 2015-16.

Covariate/category	Sample for contraceptive use analysis		Sample for birth interval analysis		Sample for antenatal visits analysis	
	Do not own	Own	Do not own	Own	Do not own	Own
**Women's age**						
15-19	4.5	1.6	0.6	0.5	4.5	2.3
20-24	16.5	11.3	21.7	17.5	33.5	28.1
25-29	20.4	18.6	42.5	42.2	36.2	39.2
30-34	17.8	19.4	22.7	26.6	16.9	20.7
35-39	15.4	18.7	8.8	10.2	6.4	7.4
40-44	13.1	16.0	2.7	2.5	1.9	1.8
45-49	12.4	14.4	0.9	0.6	0.6	0.4
**Women's schooling**						
No schooling	39.0	26.0	44.0	28.4	33.7	18.6
Up to primary	16.1	12.4	16.7	13.3	15.3	10.5
Up to secondary	40.1	46.1	36.1	47.3	45.0	51.0
More than						
secondary	4.9	15.5	3.6	11.0	6.1	19.9
**Women's age at marriage**						
<15	17.9	14.3	15.9	12.2	12.3	8.4
15-17	31.9	27.0	34.6	29.4	31.4	23.9
>=18	50.2	58.7	48.5	58.4	56.3	67.7
**Mother's parity**						
0	9.7	6.2				
1	16.4	18.5			32.3	35.6
2	30.4	37.7	38.2	46.3	33.2	36.1
3	21.1	19.8	30.4	27.9	17.7	15.4
4 or more	22.4	17.7	31.4	25.8	16.8	12.9
**Experienced child loss**						
No	86.5	88.4	80.2	81.3	87.5	89.4
Yes	13.5	11.6	19.8	18.7	12.5	10.6
**Experienced pregnancy complications**						
No	–	–	–	–	58.9	56.5
Yes	–	–	–	–	41.1	43.5
**Women's work status**						
Not working	78.6	72.2	82.4	79.4	84.8	81.2
Working	21.4	27.8	17.6	20.6	15.2	18.8
**Religion**						
Hindu	80.2	82.8	76.3	79.0	77.1	80.8
Muslim	15.4	11.4	19.5	16.5	18.5	13.9
Other	4.4	5.8	4.2	4.5	4.4	5.3
**Wealth quintiles**						
Poorest	22.6	10.5	34.9	22.0	28.8	15.0
Poorer	22.9	15.5	25.2	20.4	24.0	17.3
Middle	20.9	20.6	18.0	21.5	19.3	21.4
Richer	18.4	24.4	13.4	18.8	16.0	22.0
Richest	15.2	29.0	8.5	17.3	11.9	24.2
**Urban-rural residence**						
Urban	28.9	40.2	22.9	30.3	25.9	36.0
Rural	71.1	59.8	77.1	69.7	74.1	64.0
**Region of residence**						
South	16.0	33.0	10.7	24.2	12.7	28.1
North	11.5	14.8	11.5	15.6	11.2	15.0
Central	21.4	19.5	26.2	30.1	23.7	24.0
East	28.1	15.7	31.6	17.1	30.8	17.2
Northeast	3.5	3.0	3.6	2.9	3.9	3.4
West	19.5	14.0	16.4	10.2	12.7	12.3

All percentages are weighted.

## Footnotes

1 Geographic region of residence is categorized as “north”, “central”, “east”, “northeast”, “west”, and “south”. North consists of Chandigarh, Delhi, Haryana, Himachal Pradesh, Jammu & Kashmir, Punjab, Rajasthan, and Uttarakhand. Central consists of Chhattisgarh, Madhya Pradesh, and Uttar Pradesh. East consists of Bihar, Jharkhand, Odisha, and West Bengal. Northeast consists of Arunachal Pradesh, Assam, Manipur, Meghalaya, Mizoram, Nagaland, Sikkim, and Tripura. West consists of Dadra & Nagar Haveli, Daman & Diu, Goa, Gujarat, and Maharashtra. South consists of Andaman & Nicobar Islands, Andhra Pradesh, Karnataka, Kerala, Lakshadweep, Puducherry, Tamil Nadu, and Telangana.

2 Modern spacing includes pills, IUD, injection, diaphragm, condom, female condom, foam or jelly.

3 Modern limiting includes female sterilization and male sterilization.

4 Traditional methods include rhythm/periodic abstinence, withdrawal, lactational amenorrhea (LAM), and standard days.
